# Accuracy of MRI wall shear stress estimation

**DOI:** 10.1186/1532-429X-14-S1-W6

**Published:** 2012-02-01

**Authors:** Sven Petersson, Petter Dyverfeldt, Tino Ebbers

**Affiliations:** 1 Division of Cardiovascular Medicine, Department of Medical and Health Sciences, Linköping University, Linköping, Sweden; 2 Center for Medical Image Science and Visualization (CMIV), Linköping University, Linköping, Sweden; 3 Department of Radiology, University of California San Francisco, San Francisco, CA, USA

## Background

Wall shear stress (WSS) is a potential biomarker for vascular disease. The aim of this work was to investigate the accuracy of WSS estimation using MRI. The influence of spatial resolution, wall segmentation and voxel location were investigated over a range of WSS values by using numerical simulations.

## Methods

Three methods for WSS estimation were studied. These methods are based on 1) linear extrapolation (LE) of MRI velocity data, 2) MRI velocity data in combination with estimation of location of vessel wall, and 3) Fourier velocity encoding (FVE).

Numerical velocity fields representing axisymmetric 2D velocity profiles were generated for WSS values ranging from 1-20 N/m^2^. Based on the numerical velocity fields, phase-contrast MRI data voxels were simulated as follows: A jinc-function was used to model the 2D point spread function (PSF), and this PSF was used to obtain each voxel’s intravoxel velocity distribution. The phase-contrast MRI signal of each voxel was simulated by taking the Fourier transform of this distribution. To account for the fact that voxels cannot be positioned exactly at the wall in an MR-experiment, all simulations were carried out for ten different voxel positions uniformly distributed over one voxel length. In the LE method the spatial velocity derivative was estimated as the velocity difference between the two adjacent near-wall voxels divided by the distance between them. In the wall-based method, WSS was estimated by dividing the linear interpolated velocity at one voxel distance from the wall by the distance to the wall. Errors in segmentations of wall position were accounted for by modeling them as normally distributed with a standard deviation of 1/4 voxel size. In the FVE-based method, the WSS was obtained by first estimating the intravoxel velocity profile via a simulated FVE measurement and then computing the spatial velocity derivative near the wall. Note that the FVE-method uses larger voxels.

## Results

**Figure 1:**
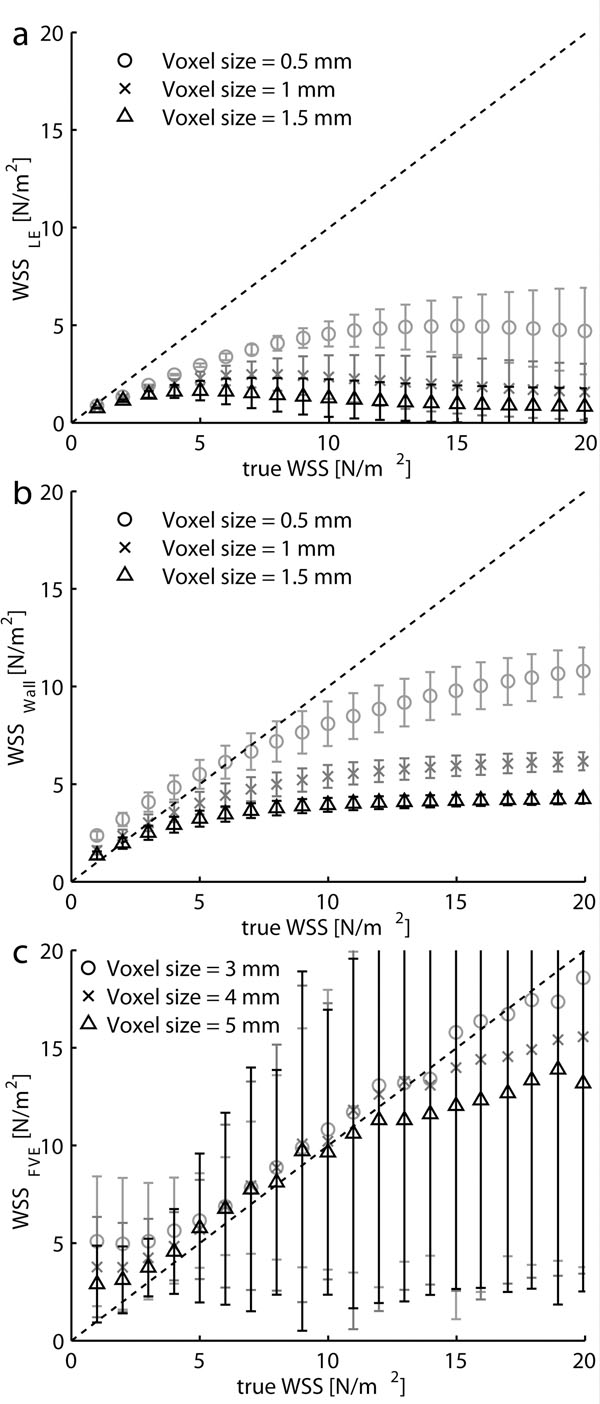
Estimates from: (a) the LE-method, WSS_LE_ (VENC = 2 m/s), (b) the wall-based method, WSS_Wall_ (VENC = 2 m/s) and (c) the FVE-method, WSS_FVE_ (velocity resolution = 0.15 m/s). The vertical axis shows the estimated WSS and the horizontal shows the true WSS. The error bars show the standard deviation due to voxel position relative the wall and segmentation (only WSS_Wall_) errors.

All methods were sensitive to spatial resolution, especially for high WSS (Fig. 1). The velocity-based methods generally underestimated the WSS, and were unable to resolve the highest WSS values (Fig. 1 a,c). The FVE-method was most sensitive to the voxel position (Fig. 1c). The wall-based method was sensitive to errors in segmentation (Fig 1 b). Linear regression results for all three methods are shown in Table 1.

**Table 1:** Linear regression results for the entire interval (1-20 N/m^2^).

Method	Voxel Size [mm]	R^2^	slope	intercept
WSS_LE_	0.5	0.44	0.19	1.87
WSS_LE_	1	0.00	0.00	1.94
WSS_LE_	1.5	0.05	-0.03	1.50
WSS_Wall_	0.5	0.82	0.43	3.17
WSS_Wall_	1	0.72	0.21	2.72
WSS_Wall_	1.5	0.62	0.12	2.36
WSS_FVE_	3	0.22	0.80	2.86
WSS_FVE_	4	0.19	0.71	2.90
WSS_FVE_	5	0.17	0.60	2.92

R^2^ is the coefficient of determination. Regression model: estimated WSS = slope*WSS + intercept.

## Conclusions

MRI WSS estimation is hampered by important limitations that require attention in studies where it is applied. WSS obtained from MR velocity data can be substantially different from WSS obtained by FVE. Although WSS was underestimated and influenced by parameter settings and segmentation errors, distinguishing areas of low and elevated WSS may be feasible.

## Funding

Swedish Research Council, Swedish Heart-Lung Foundation.

